# CyclinA1 Promoter Methylation in Self-Sampling Test

**DOI:** 10.31557/APJCP.2020.21.10.2913

**Published:** 2020-10

**Authors:** Shina Oranratanaphan, Malika Kengsakul, Surang Triratanachat, Nakarin Kitkumthorn, Apiwat Mutirangura, Wichai Termrungruanglert

**Affiliations:** 1 *Department of Obstetrics and Gynecology, Faculty of Medicine, Chulalongkorn University, Bangkok, Thailand. *; 2 *Panyananthaphikkhu Chonprathan Medical Center, Srinakharinwirot University, Nonthaburi, Thailand. *; 3 *Department of Oral Biology, Faculty of Dentistry, Mahidol University, Bangkok, Thailand.*; 4 *Center of Excellence in Molecular Genetics of Cancer and Human Diseases, Department of Anatomy, Faculty of Medicine, Chulalongkorn University, Bangkok, Thailand. *

**Keywords:** CyclinA1 promotor methylation, CCNA1, cervical cancer screening, self-sampling, CIN2+

## Abstract

**Background:**

Self sampled HPV testing is a cervical cancer screening method . However, cytology in self-sampled specimen cannot be used as a triage test. Therefore, other methods for triage should be considered. CyclinA1 (CCNA1) promoter methylation has strong association with cervical precancerous and cancerous lesion. The objective of this study was to compare the diagnostic value of CCNA1 and self-sampled specimen for detecting high-grade cervical intraepithelial lesions or worse (CIN2+).

**Materials and Methods:**

A cross sectional study was conducted. Women with abnormal cytology or positive for high risk HPV (hrHPV) indicated for colposcopic examination were enrolled. Self-collected sampling for hrHPV DNA (SS-HPV) and CCNA1 were performed. hrHPV DNA testing was done by Cobas 4800 method. CCNA1 promoter methylation was detected by CCNA1 duplex methylation specific PCR. Histopathologic result as CIN2+ obtaining from colposcopic directed biopsy or excisional procedure was considered as positive a gold standard. The results of hrHPV and CCNA1 were reported as positive or negative. Sensitivity specificity, positive predictive value, and negative predictive value of SS-HPV and CCNA1 were calculated by comparing the results with the gold standard.

**Results:**

Two hundreds and eighty women were recruited. High-grade cervical lesions and cervical cancer (CIN2+) were diagnosed in 21.8% (61 cases) of the patients. The most common type of hrHPV was non 16, 18 subtype, followed by HPV16 and 18. CCNA1 was positive in 13 patients out of whom, twelve were CIN2+. Sensitivity of CCNA1 was 19.7 % and its specificity and accuracy were 99.5% and 82.14%, respectively. The sensitivity of SS-HPV was 70.5%, and its specificity and accuracy were 39.2% and 43.3%, respectively.

**Conclusion:**

Due to high specificity and positive predictive value of CCNA1, it can be used as alarming sign of having high-grade cervical intraepithelial lesions, especially in patient who has positive hrHPV DNA test based on self-collected sampling.

## Introduction

Cervical cancer is the third most common cancer in women and the seventh one among all cancers. GLOBOCAN 528,000 new cases per year in 2012. A large majority of cervical cancer diagnoses (about 87%) are made in less developed regions (Ferlay et al., 2015). Screening programs have been proved to decrease the numbers of cervical cancer-related death, especially in developed countries. Due to high sensitivity of the primary HPV testing, this test is going to be the first line screening for cervical cancer in several countries. However, the coverage of the screening test is one of the key strategies. The Ministry of Public Health (MOPH) of Thailand reported that only 25-38% of Thai women attended cervical cancer screening program. The obstacles against cervical cancer screening in Thai women are feeling of embarrassment, fear of pain, and feeling inconvenience. Therefore, self-sampled test may increase cancer screening coverage. Self-sampled HPV DNA testing devices are simple for analysis by laboratory technicians (Bansil et al., 2014). The accuracy of self-sampled HPV test (SS-HPV) has been proved worldwide. Several studies have shown high concordance rate between clinician-sampling and self-sampling in HPV detection (Nutthachote et al., 2019; Porras et al., 2015). The advantages of SS-HPV test were ease of use, higher accessibility, and induction of less painful (Oranratanaphan et al., 2014). Although primary HPV testing for cervical screening has high sensitivity for the detection high-grade cervical lesion, the specificity and the positive predictive values are much lower than cytology (Ronco et al., 2014). Not all HPV infected women have cervical lesions. Low specificity of HPV testing can lead us to unnecessary investigation and anxiety. According to ASCCP guideline, triaging scheme for positive hrHPV testing is required. Triaging of positive HPV test from physician- collected specimen can be performed by cytology. However, many researchers reported that the accuracy of self-sampled cytology test was significantly poorer than physician- collected test (Mangold, 2019). For that reason, re-collection of specimen by the physician is recommended if triaging with cytology after positive self-sampled HPV test is required. Although triaging the positive HPV testing based on self-sampling with the same specimen is more convenience, the standard guideline for triaging with self-collected specimen is still inconclusive. Therefore, other options for triaging are required.

DNA methylation of promotor region of certain genes is known to be a cause of carcinogenesis. Recently, a strong association between cyclinA1 (CCNA1) promoter methylation and high-grade cervical lesion was reported (Chujan et al., 2014; Hansel et al., 2014). Human CCNA1 gene has been mapped to chromosome 13q12.3-q13. CCNA1 may be an important tumor suppressor gene that plays a crucial part in head and neck carcinoma and cervical cancers. In a previous study, promoter methylation of CCNA1 gene was found in 45% of tumors but in none of normal tissues, suggesting the implication of CCNA1 gene methylation in carcinogenesis (Yang et al., 2015). However, data about CCNA1 promoter methylation test in self-sampled cervical cancer screening method are not available. 

The objective of this study was to evaluate the value or ability of self-sampled CCNA1 promoter methylation (SS-CCNA1) for the detection of high-grade cervical precancerous and cancerous lesions (≥ CIN2). 

## Materials and Methods

The study was done after receiving approval form the Institutional Review Board, Faculty of Medicine, Chulalongkorn University (Protocol number 626/59). This cross-sectional study was conducted from March 1st 2017 to March 31th 2018. The sample size estimation was based on the sensitivity of CCNA1 promoter methylation for the detection of high-grade cervical lesions (Chujan et al., 2014). With α error at 0.05 and β error at 0.1, at least 250 patients were required. Additional 30 patients were recruited due to probability of patients’ dropout. The patients who attended the Colposcopic Clinic and had abnormal Pap smear or hrHPV positive were recruited. Women who underwent hysterectomy or pelvic radiation, were diagnosed with cancer, were pregnant, and those who refused to do self-sampled test were excluded. 

*Study intervention*

All patients were informed about the study objectives and methods by the researchers and trained research assistant team. After that, the patients signed the informed consent voluntarily. Before performing colposcopy, the patients were asked to perform self-collected sampling without perineum preparation. The instruction was also handed to each patient as a leaflet. The self-sampling device contained a sterile 0.5x1cm soft brush and a transport media tube. Having understood the instruction clearly, the patients placed in a comfortable position (standing or sitting). A brush was inserted about 2 inches deep into their vaginas or until it was resisted then rotated 5 times. Next, the brush was taken out and put into the transport media tube. The specimens were sent to the Department of Microbiology, Faculty of Medicine, Chulalongkorn University within 48 hours. The transport media tubes were stored at the room temperature. 

High risk HPV DNA was detected by Cobas4800. Cobas 4800 HPV test is a molecular method based on real-time PCR (RT-PCR) with a fully automated system. Cobas4800 can detect HPV16, HPV18, and other 12 high-risk HPVs (31, 33, 35, 39, 45, 51, 52, 56, 58, 59, 66, and 68 as a pool result; other hrHPV ). The result of the test will be reported as positive for hrHPV type 16, 18 or non 16, 18 other high risk . Positive of any type of hrHPV would be considered as test positive for HPV . After that, CCNA1 promoter methylation test was performed on the specimens . 

CCNA1 promoter methylation was detected by two TaqMan™ probe real-time PCRs. The CCNA1 methylation set was composed of the forward primer reverse primer and probe. The Beta actin set primers were designed in the area of no CpG island to serve as internal control. The Beta actin set consisted of forward primer, reverse primer, and probe. Both PCR reactions were prepared in a volume of 20 µL containing 10 µL of 2X TaqMan GTXpress real-time PCR master mix, 0.4 µL of 10 µmoles of each primer as well as probes and 2 µL of bisulfite-treated DNA template; the remaining volume was adjusted by adding milliQ DNase-free sterile water. Real-time PCRs were performed in duplicates using Applied Biosystem® 7500 Real-Time PCR System (Thermo Scientific™, Waltham, MA, USA). The PCR conditions were first denatured at 95 °C for 2 minutes then went after with 40 cycles as follows: denaturation at 95 °C 15 seconds, followed by annealing at 60 °C for 30 seconds. Negative control (dH20) and positive control (universal human methylated DNA (EpiTect®PCR control kit, Qaigen, USA)) were included in each PCR. A melting curve was generated to determine the specificity of the primers. Later, the threshold cycle (Ct) of the amplified methylation products was detected. The results of all samples must have Beta actin products as internal control. Data analysis reported positive if present of CCNA1 methylation product and reported negative if no CCNA1 methylation. 

The final diagnosis was achieved from histopathologic results considered as gold standard. Cervical tissue biopsy and/or endocervical curettage and/or endometrial sampling was performed by an experienced colposcopist who had passed the colposcopic training course and the 50 validated cases before performing colposcopic examination in this study. Those without significant colposcopic findings were also randomly biopsied at 6 and 12 o’clock positions. Histopathological diagnosis was reported according to WHO’s guidelines (2014) by gynecologic pathologists. The most severe histopathological result from cervical biopsies, conization, or hysterectomy was used as the gold standard.

The statistical analysis was performed using SPSS version 22 (SPSS Inc., Chicago, IL, USA). All information was analyzed as mean and percentage. Final histological results from colposcopic directed biopsy or excisional procedure were considered as gold standard. Histologic result included benign tissue was classified as low-grade cervical lesions (CIN 2-) and if included HPV effect or CIN1 was considered as negative in gold standard. High-grade cervical lesions (≥ CIN2) included CIN2, CIN3, and malignancy. CIN2+ was considered as positive in gold standard. CCNA1 and hrHPV testing results were reported as positive or negative. The diagnosis performance of self-sampled HPV test and self-sampled CCNA1 promotor methylation test for the detection of cervical precancerous and cancerous lesion (≥ CIN2) were calculated in respect to sensitivity, specificity, PPV, NPV, accuracy, likelihood ratio, and 95% confidence interval. 

## Results

The study was conducted from March 1st 2017 to March 31th 2018. Two hundred and eighty women were recruited from the Colposcopy Clinic, King Chulalongkorn Memorial Hospital. The mean age of the patients was 44.8 years (SD11.2). The majority of the patients were premenopause (68.2%).

Cobas 4800 HPV testing was performed on all 280 patients. The percentage of hrHPV DNA detection and cytology results are shown in Table 1. The most severe histopathological result from cervical biopsies, conization, and hysterectomy was used as the gold standard. The histopathological results are shown in percentage in Table 1. The histopathological results were divided in two groups of low-grade cervical intraepithelial lesion and high-grade cervical intraepithelial lesion. Low-grade cervical intraepithelial lesion or CIN2- was 78.21% (219) and high-grade cervical intraepithelial lesion or ≥ CIN2 was 21.79% (61). Notably, hrHPV DNA was detected in 133 out of 219 samples in low-grade cervical lesion (<CIN2) group and 43 out of 61 samples in high-grade cervical lesion (≥CIN2) group. CCNA1 promoter methylation was detected in 12 out of 61 high-grade cervical lesion samples (≥CIN2) and only one methylation was detected in less severe group (<CIN2). Details of results on CCNA1, hrHPV DNA testing, and cytology in the samples with <CIN2 and ≥CIN2 are shown in Table 2. 

The diagnostic performance of CCNA1 promotor methylation and hrHPV DNA in self-sampled test for high-grade cervical lesion are presented in Table 3. The sensitivity of self-sampled CCNA1 promoter methylation test was lower than that of HPV DNA test (19.67% vs. 70.49%). The specificity and accuracy of SS-CCNA1 promoter methylation test were much higher than those of SS-HPV DNA (99.54% vs. 39.2% and 82.14% vs. 43.3%, respectively). 

All of the CCNA1 positive cases also had positive HPV test, 6 of them were positive for non 16, 18 other high risk subtype; other 6 of them were positive for type 16 and one of them was positive for type 18. From 61 patients with high grade lesion, CCNA1 was positive in 12 ones. However, CCNA1 was negative in 218 patients out of 219 ones of negative gold standard , True negative value of CCNA1 was very high. From 13 CCNA1 positive patients, 10 were CIN2 or CIN3. The rest of them were squamous cell carcinoma, adenocarcinoma, and benign. This means that 12 out of 13 positive cases had significant lesions. The result of tests is shown in Table 2. 

**Table1:** Baseline Characteristic and Information of the Study Population, Including Menopausal Status and Cervical Cancer Screening Results (N=280)

	N (%)
Menopausal status	
Premenopausal	191 (68.2)
Postmenopausal	89 (31.8)
Cytology	
NILM with hrHPV	14 (5.0)
ASC-US	86 (30.7)
ASC-H	27 (9.6)
LSIL	98 (35.0)
HSIL	32 (11.4)
Squamous cell carcinoma	8 (2.9)
Adenocarcinoma	15 (5.4)
HPV genotype	
HPV16	46 (16.4)
HPV18	12 (4.3)
Other hrHPV	145 (51.8)
More than 1 type	18 (6.4)
Histopathological results	
Benign	38 (13.6)
CIN1	181 (64.6)
CIN2-3	49 (17.5)
Squamous cell carcinoma	5 (1.8)
Atypical glandular cell	7 (2.5)

CIN, cervical intraepithelial neoplasia; NILM, Negative for intraepithelial lesion; ASC-US, Atypical Squamous Cell Undetermined Significance; ASC-H, Atypical Squamous cell cannot exclude HSIL; LSIL, Low-grade squamous intraepithelial lesion; HSIL, High-grade squamous intraepithelial lesion; SCC, squamous cell carcinoma; AGC, atypical glandular cell; hrHPV, high risk human papilloma virus

**Table 2:** Cytology, hrHPV, and CCNA1 Testing Results in CIN2- and >CIN2 Groups

Final Pathology	Cytology (N)		HPV (N)						CCNA1 (N)	
			Neg	Any	16	18	other	>1	Neg	Pos
CIN2-(N= 219)	NILM	12	86	133	28	8	119	16	218	1
	ASC-US	77								
	ASC-H	18								
	LSIL	89								
	HSIL	13								
	SCC	0								
	AGC	10								
CIN 2 or more (N= 61)	NILM	2	18	43	17	4	26	2	49	12
	ASC-US	9								
	ASC-H	9								
	LSIL	9								
	HSIL	19								
	SCC	8								
	AGC	5								

NILM, Negative for intraepithelial lesion; ASC-US, Atypical Squamous Cell Undetermined Significance; ASC-H, Atypical Squamous cell cannot exclude HSIL; LSIL, Low-grade squamous intraepithelial lesion; HSIL, High-grade squamous intraepithelial lesion; SCC, squamous cell carcinoma; AGC, atypical glandular cell; Other, non16, 18 other high rish HPV subtype, >1: more than 1 HPV subtypes; Neg, negative; Pos, positive

**Table 3:** The Diagnostic Value Self- of Sampled hrHPV DNA and CCNA1 Promoter Methylation for Detection of CIN2+

	Sensitivity	Specificity	PPV	NPV	Accuracy	Positive LR	Negative LR
	% (95%CI)						
HPV	70.5 (58.0-80.0)	39.3 (33.0-46.0)	24.43 (18.7-31.0)	82.69 (74.0-89.0)	46.1 (40.0-51.0)	1.2	0.7

CCNA1 methylation	19.7 (11.6-31.1)	99.54 (97.5-99.9)	92.3 (66.7-98.6)	81.6 (76.6-85.8)	82.1 (77.2-86.2)	42.76	0.8

PPV, positive predictive value; NPV, negative predictive value; LR, likelihood ratio

## Discussion

Primary hrHPV testing is becoming the first line screening for cervical cancer because of its high efficacy and cost effectiveness (Skroumpelos et al., 2019; Termrungruanglert et al., 2017). Primary hrHPV testing specimen collection can be performed both by clinician and the patients. Self-sampling can reduce the barriers to cancer screening such as embarrassment, fear of pain, or fear of speculum examination (Oranratanaphan et al., 2014). Self-sampling for cervical cancer screening has has enough accuracy and reliability for the detection of hrHPV. The performance of hrHPV DNA test in self-sampling devices was reported to be comparable with clinician-sampling (Nutthachote et al., 2019; Porras et al., 2015). According to previous studies, sensitivity of self-sampled HPV test ranges between 84-100% (Bansil et al., 2014; Porras et al., 2015). The sensitivity of self-sampled hrHPV for the detection of high-grade cervical lesion in this study was 70.49%, which was slightly lower than previous reports. 

HPV DNA testing has very high sensitivity but low specificity that can cause some problems such as overinvestigation and anxiety of the patients. Theoretically, not all women with HPV infection progress to cervical cancer. Approximately 70-80% of HPV infections will resolve spontaneously. Approximately 20% progress to precancerous lesions and only a few of them progress to cancerous lesion. Therefore, not all women with a positive hrHPV test develop lesions . Current recommended triage strategies for primary HPV screening include HPV partial genotyping with cervical cytology and alternatively host and viral methylation (Wentzensen et al., 2016). However, self-samples are not suitable for cytology. Therefore, molecular testing is considered for a triage model. 

Recent evidence has demonstrated that methylation of cyclinA1 (CCNA1) gene may also considerably conduce to the process of tumorigenesis (del Mistro et al., 2017). It has been widely described in several studies that CCNA1 may be an important tumor suppressor gene. In a previous study, CCNA1 was proven to have high distinctive ability to differential benign or low-grade cervical intraepithelial lesion from high-grade intraepithelial lesion (Chujan et al., 2014; Yang et al., 2010). This study found CCNA1 gene methylation positive 12 cases in high- grade lesion and positive one case in benign specimen, which was correlated with Yang et al.’s findings reporting CCNA1 can be positive 11.1 % in normal cervix, 25% in LSIL, 55.6% in HSIL and 80% in cervical cancer (Yang et al., 2014). 

Sensitivity of high-grade cervical lesion detection in self-sampled CCNA1 promoter methylation test in this study was quite low (19.67%), but its specificity was as high as 99.54% and its accuracy was 82.14%. The sensitivity obtained in our study was in contrast with the findings of a study in Chiangmai, a northern city in Thailand (Chujan et al., 2014). They reported a high sensitivity and specificity for CCNA1 methylation in detection of high-grade cervical lesions, revealing sensitivity and specificity of 83% and 96.88%, respectively. We hypothesized that different methods of specimen collection may have an influence on the detection rate of CCNA1 methylation. Chujan et al., compared HPV testing and CCNA1 promoter methylation in terms of diagnostic performance in detection of high-grade cervical lesions based on cytology. However, the sensitivity and specificity of CCNA1 were compatible to our previous study that was performed on clinician-collected specimen (Oranratanaphan et al., 2020). Based on the result of this study, CCNA1 positive was a strong marker for having high-grade intraepithelial lesions in the patient. This result was correlated with that of Kitkumthorn et al.’s study, reporting a strong association between CCNA1 promoter methylation and 3 (Kitkumthorn et al., 2006). This study was performed to evaluate the role of CCNA1 in self-sampling method. This study had some limitations. Cost effectiveness of was not evaluated in this study, and long-term follow up of the patients especially those who had CCNA1 positive but their histological result was benign was not performed. 

In conclusion, we found that self-sampled HPV DNA testing had high sensitivity but low specificity that may cause women do unnecessary interventions such as colposcopic directed biopsy. Due to the low sensitivity of CCNA1 promoter methylation, it should not be used as screening method but its high specificity is valuable to be used as an alert sign of having high-grade lesion. Cytology cannot be used to triage the patients who have positive HPV in self-sampling method. However, CCNA1 methylation may be used as a triage test in SS-HPV positive test. Further studies are still required .
